# Spin-thermoelectric effects in a quantum dot hybrid system with magnetic insulator

**DOI:** 10.1038/s41598-022-09105-z

**Published:** 2022-03-30

**Authors:** Piotr Trocha, Emil Siuda

**Affiliations:** Institute of Spintronics and Quantum Information, Faculty of Physics, Adam Mickiewicz University, Poznań, 61-614 Poland

**Keywords:** Physics, Condensed-matter physics

## Abstract

We investigate spin thermoelectric properties of a hybrid system consisting of a single-level quantum dot attached to magnetic insulator and metal electrodes. Magnetic insulator is assumed to be of ferromagnetic type and is a source of magnons, whereas metallic lead is reservoir of electrons. The temperature gradient set between the magnetic insulator and metallic electrodes induces the spin current flowing through the system. The generated spin current of magnonic (electric) type is converted to electric (magnonic) spin current by means of quantum dot. Expanding spin and heat currents flowing through the system, up to linear order, we introduce basic spin thermoelectric coefficients including spin conductance, spin Seebeck and spin Peltier coefficients and heat conductance. We analyse the spin thermoelectric properties of the system in two cases: in the large ondot Coulomb repulsion limit and when these interactions are finite.

## Introduction

Nowadays, new and environmentally friendly sources of energy are desirable. One of such a possibility is given by conversion of waste heat generated by electronic devices to useful electric power. Waste heat is produced by every electronic device through which electric current is passed due to coupling between electrons and phonons. The main source of waste heat is associated with Joule heating, however in the semiconductors also other mechanisms are responsible for heat dissipation. Moreover, the desired miniaturization of electronic devices leads to even greater problems with excessive heat generation which reduces functionality of a device. Although many attempts have been undertaken in order to reduce the generated heat or effectively dissipate it to the environment, the practical usage of such waste heat seems to be at the beginning of the road. Thus, efficient ways for conversion of heat to electric energy are desired. Although thermoelectricity has been known for a long time, it wasn’t broadly utilized due to low efficiency present in conventional materials. Recently, it turned out that the effects resulting from reduction of dimensionality can lead to increase of thermoelectric efficiency giving possibilities to create efficient heat-to-electric power converters^1–4^. Specifically, quantum dots seem to be good candidates for high-efficiency energy-converters as they reveal level and charge quantization which strongly affects the thermoelectric properties which has been shown theoretically^5–16^ and observed in experiments^17–21^. Moreover, quantum interference effects in double quantum dots can additionally lead to large enhancement of thermoelectric response^22^. Apart from that, a two-site nanostructure attached to two conducting leads and connected to a phonon bath can exhibit a large thermopower and high figure of merit^23^.

Furthermore, discovery of spin Seebeck effect in metallic magnets^24^ renewed research interest in the field of so-called spin caloritronics which describes interaction of spins with heat currents. Its main goal is to utilize dissipated heat energy to drive spin currents which can be realized by using temperature gradient instead of voltage bias^25^. It also quickly resulted in the discovery of various spin counterparts of thermoelectric phenomena^26–28^, including spin-dependent Peltier effect^29^ and spin Peltier effect^30^. The latter effect can be explained by conversion of spin current of electronic type induced in metal to magnon heat current in a magnetic insulator by means of spin transfer torque. Conversely, a temperature difference between magnetic insulator and metallic electrode may lead to thermal pumping of spin current i.e. can convert a heat flow into a spin voltage resulting in the spin Seebeck effect^31^. This spin current is further transformed into an electric voltage by means of the inverse spin Hall effect^32–34^.

Generation of spin thermoelectric effects by means of spin waves seems to be additionally promising as magnons are carriers of information without the drawback of generating waste heat. This is because magnons carry no charge, but only angular momentum and energy and they can propagate long distances without scattering^35–37^. Recently, magnons have been utilized in many well-known devices, including multiplexers^38^, diodes^39,40^, transistors^41^ and logic devices^42^. Moreover, rectification of thermally generated spin current and negative differential spin conductance have been proposed in magnon tunnel junction under temperature bias^43,44^.

Another possibility of conversion of spin waves to electronic spin current and vice versa has been studied in a hybrid system involving both metallic and magnonic reservoir^45–47^. Efficient conversion of spin current can be also achieved by coupling the magnetic insulator and metallic electrodes through a quantum dot^48,49^.

Spin thermoelectric effects have been also observed in antiferromagnetic hybrid systems. Specifically, thermal generation of spin current from the insulating antiferromagnets through the longitudinal spin Seebeck effect has been reported^50^ and described theoretically^51^. Thermally generated spin transport in magnetic multilayered structures consisting of nonmagnetic metals, antiferromagnetic insulators and/or ferromagnetic insulators has been recently studied^52–55^. Moreover, a large enhancement of thermally generated spin current has been reported in a hybrid system with normal metal, antiferromagnetic and ferromagnetic insulators layered structure^56^. Apart from that, giant magneto-spin-Seebeck effect has been predicted in all-insulating spin valve with antiferromagnetic insulator sandwiched between two ferromagnetic insulator layers^57^.

**Figure 1:**
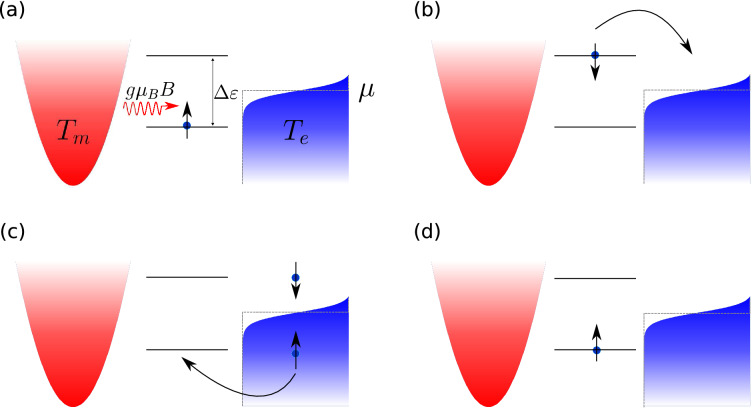
Schematic picture showing the idea of converting magnon current to electron spin current by means of temperature difference. The red parabola symbolizes the magnon reservoir, whereas blue curve stands for density of electrons in the metallic lead. The blue area below the curve denotes states occupied by electrons and the white space above the curve are empty states. Furthermore, red color is associated with higher temperature than blue one, i.e. $T_m>T_e$. Zeeman split dot’s energy level is depicted by two black solid horizontal lines. The splitting of dot’s energy level equals $\Delta \varepsilon =\varepsilon _{\downarrow }-\varepsilon _{\uparrow }=g\mu _B B$. Magnon, carrying energy $g\mu _B B$, is depicted as red wavy arrow, whereas blue dot with vertical arrow denotes an electron with a given spin.

In the present paper we investigate spin thermoelectric effects in a system consisting of a quantum dot coupled to magnetic insulator and metallic leads. Magnetic insulator is a source of magnons, whereas magnetic metal is a reservoir of electrons. In turn, the QD works as a converter of spin current of magnonic type to spin current of electronic type and vice versa. The process of converting magnon current to electron spin current by means of temperature difference set between the two leads is schematically drawn in Fig. 1 and can be understood as follows. Assume that the whole system is placed in a magnetic field *B* directed opposite to the *z*-axis and the dot level is split due to this field $\varepsilon _{\downarrow ,\uparrow }=\varepsilon _d\pm g\mu _B B/2$. For the sake of simplicity, assume that intradot Coulomb repulsion is infinitely large (then the dot can be occupied at most by one electron) and dot’s bare level $\varepsilon _d$ is placed at the chemical potential of metallic lead $\mu$. Therefore, QD is occupied by an electron with spin up orientation as $\varepsilon _{\uparrow }<\mu$. Next, assume that temperatures of magnetic insulator and metallic electrodes are set to be $T_m>T_e$. In this situation, magnons flow from the magnonic reservoir to the dot. Absorption of a magnon by QD excites the spin-$\uparrow$ electron which is simultaneously accompanied by (its) spin-flip process. As a result, spin-$\downarrow$ electron of energy $\varepsilon _{\downarrow }$ can flow from the QD to metallic lead emptying it. Furthermore, an electron with spin $\uparrow$ can tunnel from metallic lead to the dot. Thus, the magnon current flowing from the magnonic reservoir is converted into pure spin current of electronic type in the metallic electrode. On the other hand, when the temperature of the electronic reservoir is higher than that of the magnonic one, $T_m < T_e$, the spin-flip processes on the dot excite magnons in the magnonic reservoir which is associated with conversion of spin current of electronic type to magnon current.

The paper is organized in the following way: section “Theoretical description” contains the theoretical description of the considered system and it is divided into three parts. In the first part we describe the details of the model. The second part is devoted to the derivation of electron and heat current formula, whereas in the third part we introduce linear response theory for spin thermoelectric effects. In section “Results and discussion” we describe the obtained results and provide the discussion of them both for large *U* limit and for case of finite *U*. Finally, we provide short conclusion section.

## Theoretical description

### Model Hamiltonian

The system taken into consideration consists of a single-level quantum dot (QD) attached to magnetic insulator (MI) and metallic electrodes and is schematically presented in Fig. 1. The system is under the influence of an external magnetic field *B*. The system is modeled by Hamiltonian of the form:

1 $$\begin{aligned} H=H_{\mathrm{e}}+H_{\mathrm{QD}}+H^{\mathrm{t}}_{\mathrm{e}}+H_{\mathrm{m}}+H^{\mathrm{t}}_{\mathrm{m}}, \end{aligned}$$

where the first term, $H_{\mathrm{e}}=\sum _{{\mathbf {k}}\sigma }\varepsilon _{{\mathbf {k}}\sigma }c_{{\mathbf {k}}\sigma }^{\dagger }c_{{\mathbf {k}}\sigma }$, describes electrons in the left metallic lead. Here, $\varepsilon _{{\mathbf {k}}\sigma }$ is the single-particle energy of an electron with a wavevector ${\mathbf {k}}$ and spin $\sigma =\uparrow ,\downarrow$. The second term describes a single-level quantum dot and acquires the form:

2 $$\begin{aligned} H_{\mathrm{QD}}=\sum _{\sigma }\varepsilon _{d\sigma }d_{\sigma }^{\dagger }d_{\sigma }+Un_{\uparrow }n_{\downarrow }, \end{aligned}$$

with $\varepsilon _{d\sigma }=\varepsilon _{d}-{\hat{\sigma }}g\mu _BB/2$ denoting the dot’s level energy. The dot’s degeneracy is lifted by an external magnetic field *B* ($\varepsilon _{d}$ is the bare dot’s level energy). Here, *g* is the Lande factor of the dot, $\mu _B$ is the Bohr magneton, while ${\hat{\sigma }}=+(-)$ for $\sigma =\uparrow (\downarrow )$. The second term in (2) refers to the intradot Coulomb repulsion between electrons of opposite spins with *U* being the relevant Hubbard parameter. Tunneling of electrons between the QD and metallic lead is described by:

3 $$\begin{aligned} H^{\mathrm{t}}_{\mathrm{e}}=\sum _{{\mathbf {k}}\sigma }V_{{\mathbf {k}}\sigma }c_{{\mathbf {k}}\sigma }^{\dagger }d_{\sigma }+ \mathbf {{H.c.}}, \end{aligned}$$

where $V_{{\mathbf {k}}\sigma }$ are the corresponding tunneling matrix elements.

Magnetic insulator is described by the $H_{\mathrm{m}}$ in (1) which is modeled by Heisenberg Hamiltonian restricted to the nearest neighbours interactions;

4 $$\begin{aligned} H_{\mathrm{m}}=-J_{\mathrm{ex}}\sum _{\langle i,j\rangle }{\mathbf {S}}_{i}\cdot {\mathbf {S}}_{j} -g_m^\alpha \mu _BB\sum _{i}S_{i}^z. \end{aligned}$$

Here, $\langle i,j\rangle$ denotes summation over nearest neighbours, $J_{\mathrm{ex}}$ ($J_{\mathrm{ex}}>0$) is the corresponding nearest-neighbour exchange integral, while $g_m$ is the Lande factor of the magnetic insulator. Note that Lande factors for QD and magnetic insulator differ. Due to the large tunability of quantum dots, one can meet the criterion $g\ge g_m$ which allows for nonzero magnon current. This condition is essential from the point of view of magnon filtering as only magnons with energy equal to the Zeeman splitting of the dot level can be transferred through the QD i.e. only when the equality holds, $\varepsilon _{\mathbf {q}}=\Delta \varepsilon$ with $\Delta \varepsilon = g\mu _B B$. Note also that QD and magnetic insulator are placed in the same magnetic field *B*. In the following we assume $B>0$ as only in this case both energy and angular momentum conservation can be obeyed when absorbing/emitting a magnon. Introducing the operators $S_{\alpha i}^{\pm }=S_{\alpha i}^{x}\pm iS_{\alpha i}^{y}$ and performing the Holstein–Primakoff transformation^58^: $S_{i}^{+}= \sqrt{2S-a_{i}^{\dagger }a_{i}}a_{i}$, $S_{i}^{-}= a_{i}^{\dagger }\sqrt{2S-a_{i}^{\dagger }a_{i}}$ and $S_{i}^{z}= S-a_{i}^{\dagger }a_{i}$. Assuming that $\langle a_{i}^{\dagger }a_{i}\rangle /(2S)\ll 1$ one can expand the square roots and rewrite the Hamiltonian (4) in the Fourier space taking only quadratic terms as follows;

5 $$\begin{aligned} H_{\mathrm{m}}=\sum _{{\mathbf {q}}}\varepsilon _{{\mathbf {q}}}a_{{\mathbf {q}}}^{\dagger }a_{{\mathbf {q}}}, \end{aligned}$$

where $\varepsilon _{{\mathbf {q}}}$ is the spin wave energy for the wavevector $\mathbf{q}$ and acquires the form, $\varepsilon _{{\mathbf {q}}}=2zSJ_{ex}(1-\gamma _{{\mathbf {q}}})+g_m\mu _BB$. Here, *z* denotes the number of nearest neighbors and $\gamma _{{\mathbf {q}}}=1/z\sum _{\mathbf {\delta }_l}{\mathrm {e}^{i{\mathbf {q}}\cdot \mathbf {\delta }_l}}$ is a geometric factor that depends on the crystal structure where the sum is over the position vectors $\mathbf {\delta }_l$ of the nearest neighbors. Note that condition $g\ge g_m$ becomes clear when one considers the above dispersion relation together with energy conservation $\varepsilon _{\mathbf {q}}=\Delta \varepsilon$. Apart from that, we neglect higher-order terms of the expansion which can lead to temperature dependence of the magnonic dispersion relation. These corrections are small for relatively low temperatures, and thus, can be neglected^59^. For completeness, we also neglect variation of the spontaneous magnetization of magnetic insulator with temperature. This is justified as long as we assume a low temperature regime and take into account the relatively small range of temperature in our considerations^60^.

The last term in Eq. (1) describes exchange coupling between the quantum dot and the magnetic insulator and making the same procedure, as for magnetic insulator Hamiltonian, it can be expressed as;

6 $$\begin{aligned} H^{\mathrm{t}}_{\mathrm{m}} = \sum _{{\mathbf {q}}}j_{{\mathbf {q}}}a_{{\mathbf {q}}}^{\dagger }d_{\uparrow }^{\dagger }d_{\downarrow }+\mathbf { \mathrm {H.c.}}, \end{aligned}$$

where $j_{{\mathbf {q}}}$ depends generally on the distribution of interfacial spins and on coupling between these spins and the quantum dot. Here, this coupling will be treated as a parameter.

### Method

In order to calculate spin current generated by the temperature difference between magnonic reservoir (magnetic insulator) and metallic electrode we employ Pauli’s master equation method which correctly describes the transport properties for weak coupling regime. Thus, we assume that the couplings with external electrodes are treated perturbatively. The master equations in the stationary limit can be written as;

7 $$\begin{aligned} \sum _{j}\left( W_{ji}P_{j}-W_{ij}P_{i}\right) =0, \end{aligned}$$

with $W_{ij}$ denoting the transition rate from the dot’s state $|i\rangle$ with energy $E_{i}$ to the state $|j\rangle$ with energy $E_{j}$. Moreover, to ensure normalization condition we introduce, $\sum _{i}P_{i}=1$. The transition rates are determined by Fermi’s Golden Rule and can be written in the form;

8 $$\begin{aligned} W_{ij}=\sum _{\alpha =e,m}\sum _{\gamma =+,-} W_{ij}^{\alpha ,\gamma }, \end{aligned}$$

with $\alpha =e$ denoting rate associated with tunneling of electrons, whereas $\alpha =m$ corresponds to magnon rate. The electron tunneling rates acquire the form;

9 $$\begin{aligned} W_{ij}^{e,+}= & {} \frac{1}{\hbar }\Gamma _{e}^{\sigma }|\langle j|d_{\sigma }^{\dag }|i\rangle |^{2}f_{\sigma }^{+}(E_j-E_i), \nonumber \\ W_{ij}^{e,-}= & {} \frac{1}{\hbar }\Gamma _{e}^{\sigma }|\langle j|d_{\sigma }|i\rangle |^{2}f_{\sigma }^{-}(E_i-E_j), \end{aligned}$$

where $f_{\sigma }^{+}(\varepsilon ) = 1/[\exp (\frac{\varepsilon -\mu _{\sigma }}{k_BT_{e}})+1]$ is the Fermi–Dirac distribution function with $\mu _{\sigma }$ being the electrochemical potential in the metallic electrode $\alpha =e$ for spin $\sigma$, while $T_e$ is the corresponding temperature. Furthermore, $f_{\sigma }^{-}(\varepsilon )\equiv 1-f_{\beta \sigma }^{+}(\varepsilon )$ and $\Gamma _{e}^{\sigma }$ denotes tunneling strength between metallic lead and dot which is assumed to be independent on energy in accordance with wide band approximation. This allows us to parametrize the coupling strength as $\Gamma _{e}^{\sigma } = 2\pi \langle |V_{\mathbf{k}\sigma }|^{2}\rangle \rho _{e}=\Gamma _{e}$ for $\sigma =\uparrow ,\downarrow$, where $\langle |V_{\mathbf{k}\sigma }|^{2}\rangle$ is the corresponding average over $\mathbf{k}$ and $\rho _{e}$ stands for the density of electron states in the metallic lead $\alpha =e$. In turn, the magnonic tunneling rates are nonzero only for transitions between the dot’s states $|\uparrow \rangle$ and $|\downarrow \rangle$ and are given by;

10 $$\begin{aligned} W_{\sigma {\bar{\sigma }}}^{m,{\tilde{\sigma }}}=\frac{1}{\hbar }\Gamma _m|\langle {\bar{\sigma }}|d_{{\bar{\sigma }}}^{\dag }d_{\sigma }|\sigma \rangle |^{2}n^{{\tilde{\sigma }}}[{\tilde{\sigma }}(E_{{\bar{\sigma }}}-E_{\sigma })], \end{aligned}$$

where ${\tilde{\sigma }}=+1$ for $\sigma =\uparrow$ and ${\tilde{\sigma }}=-1$ for $\sigma =\downarrow$. Here, $n^{+}(\epsilon )$ is the Bose-Einstein distribution function, $n^{+}(\epsilon ) = 1/[\exp (\frac{\epsilon }{k_BT_{m}})-1]$ and $n^{-}(\epsilon )\equiv n^{+}(\epsilon )+1$. Apart from that, $\Gamma _m$ stands for coupling strength between dot and magnonic reservoir and can be written as $\Gamma _{m} = 2\pi \langle |j_{\mathbf{q}}|^{2}\rangle \rho _{m}$ with $\rho _{m}$ being density of magnon states in the insulating lead and $\langle |j_{\mathbf{q}}|^{2}\rangle$ denoting relevant average. Generally, the density of magnon states reveals non-trivial dependence on the energy. Oppositely to the transport of electrons, for which only states within the range $k_BT$ around Fermi level are crucial and thus, the density of states can be regarded as flat, in the case of bosons (here magnons) wide band approximation doesn’t work in general and explicit energy dependence of density of states should be considered. However, in the case of (two-dimensional) yttrium iron garnet structure its density of states can be considered constant in the relatively large range of energy^61^. Employing this feature we assume energy independent coupling strength with a magnonic reservoir.

After calculating relevant transition rates by using Eq. (8) and corresponding probabilities using Eq. (7) we obtain magnon current flowing from magnonic reservoir to the dot defined by the formula;

11 $$\begin{aligned} J_{\mathrm{m}} = \left( P_{\uparrow }W_{\uparrow \downarrow }^{m,+}-P_{\downarrow }W_{\downarrow \uparrow }^{m,-}\right) , \end{aligned}$$

whereas spin current flowing from magnonic electrode is given by $J_{\mathrm{s}}^{m}=-\hbar J_{\mathrm{m}}$. As we assumed the case of $B>0$ then each magnon caries the spin angular momentum with the *z* component equal to $-\hbar$, the magnon current $J_{\mathrm{m}}$, defined as the number of magnons transmitted from magnonic reservoir to the QD in a unit time, is equal to the corresponding spin current divided by $-\hbar$. Therefore, the spin current and magnon current have opposite signs. In turn, spin current flowing from metallic electrode is determined by angular momentum conservation, $J_{\mathrm{s}}^{e}=-J_{\mathrm{s}}^{m}$. $J_{\mathrm{s}}^{e}$ can be directly expressed by means of corresponding charge currents flowing in two spin channels in electronic reservoir, i.e. $J_{\mathrm{s}}^{e}= \frac{\hbar }{2e}(I_{\uparrow }^{e}-I_{\downarrow }^{e})$. As no net current can flow through the system one concludes that, $I_{\uparrow }^{e}+I_{\downarrow }^{e}=0$. Finally, one derives the following formula for spin current flowing from magnonic reservoir to electronic one relevant for $U\rightarrow \infty$^49^;

12 $$\begin{aligned} J_{\mathrm{s}}=-\frac{\Gamma _{m}\Gamma _{e}^{\uparrow }\Gamma _{e}^{\downarrow }\left[ f_{\downarrow }^{+} \left( f_{\uparrow }^{+}-1\right) +n_{m}^{+}\left( f_{\uparrow }^{+}-f_{\downarrow }^{+} \right) \right] }{\Gamma _m\Gamma _{e}^{\downarrow }\left[ f_{\downarrow }^{+}+n_{m}^{+}\left( 1+f_{\downarrow }^{+}\right) \right] +\Gamma _{m}\Gamma _{e}^{\uparrow }\left[ 1+n_{m}^{+}\left( 1+f_{\uparrow }^{+}\right) \right] +\Gamma _{e}^{\uparrow }\Gamma _{e}^{\downarrow }\left( 1- f_{\downarrow }^{+}f_{\uparrow }^{+}\right) }, \end{aligned}$$

where $f_{\uparrow }^{+} =f_{\uparrow }^{+}(\varepsilon = \varepsilon _\uparrow )$, $f_{\downarrow }^{+} =f_{\downarrow }^{+}(\varepsilon = \varepsilon _\downarrow )$, and $n_{m}^{+} = n_{m}^{+}(\epsilon = g\mu _BB)$. In turn, the heat current associated with magnonic current is given by

13 $$\begin{aligned} J_{\mathrm{Q}}=(\varepsilon _{\downarrow }-\varepsilon _{\uparrow })J_{\mathrm{m}}=g\mu _B BJ_{\mathrm{m}}, \end{aligned}$$

Similarly, one can obtain the formulas for spin and heat currents for finite values of parameter *U*. However, we don’t present them here as they acquire more complex forms.

### Spin thermoelectric effects–linear response theory

Previously, we introduced spin-dependent chemical potential $\mu _{\sigma }$ in the metallic lead which may be induced by spin accumulation or may result from externally applied spin bias. The spin bias, $V_s$ is given by $eV_s\equiv \Delta \mu _s=\mu _{\uparrow }-\mu _{\downarrow }$. Thus, one can write;

14 $$\begin{aligned} \mu _{\sigma }=\mu \pm \frac{\Delta \mu _s}{2}, \end{aligned}$$

where upper (lower) sign corresponds to $\sigma =\uparrow$ ($\sigma =\downarrow$). Generally, the temperatures associated with two spin channels can be different. Here, we neglect this effect and assume the same temperature for both spin species, i.e. $T_{e}^{\uparrow }=T_{e}^{\downarrow }\equiv T_e$. Furthermore, we parametrize the temperatures in metallic and magnonic reservoir by $T_{\alpha }=T\pm \Delta T/2$, where upper (lower) sign corresponds to $\alpha =m$ ($\alpha =e$) and $\Delta T=T_m-T_e$ is temperature bias.

Assuming that temperature and spin biases are small, i.e. for $\Delta T\ll T$ and $\Delta \mu _s\ll \mu$ one expands magnon (spin) and heat currents, Eqs. (12) and (13), up to linear order and obtains;

15 $$\begin{aligned} \left( \begin{array}{c} J_m \\ J_Q \\ \end{array} \right) = \left( \begin{array}{cc} G_s &{} L_s T \\ L_s T &{} \varkappa _s T \\ \end{array} \right) \left( \begin{array}{c} \Delta \mu _s \\ \Delta T/T \\ \end{array} \right) , \end{aligned}$$

where $G_s$ is spin conductance which for $U\rightarrow \infty$ acquires the following form;

16 $$\begin{aligned} G_s=\frac{1}{\hbar }\frac{\Gamma _m\Gamma _e^\uparrow \Gamma _e^{\downarrow }{\mathscr {F}}(\varepsilon _{\uparrow }+\varepsilon _{\downarrow }+\mu )}{[{\mathscr {F}}(\varepsilon _{\uparrow }+\varepsilon _{\downarrow })+{\mathscr {F}}(\varepsilon _{\uparrow }+\mu )+{\mathscr {F}}(\varepsilon _{\downarrow }+\mu )] [\Gamma _m(\Gamma _e^{\uparrow }+\Gamma _e^{\downarrow }){\mathscr {F}}(\mu )+\Gamma _e^{\uparrow }(\Gamma _m+2\Gamma _e^{\downarrow }){\mathscr {F}}(\varepsilon _{\downarrow }) +\Gamma _e^{\downarrow }(\Gamma _m-2\Gamma _e^{\uparrow }){\mathscr {F}}(\varepsilon _{\uparrow })]} \end{aligned}$$

with ${\mathscr {F}}(x)=k_BT\exp {(x/k_BT)}$. Apart from that, $\varkappa _s=(\varepsilon _{\downarrow }-\varepsilon _{\uparrow })L_s$ is magnetic contribution to heat conductance (in the absence of spin bias, i.e. when $\Delta \mu _s=0$) and $L_s=\frac{\varepsilon _{\downarrow }-\varepsilon _{\uparrow }}{T}G_s$. Note that the above linear response matrix reflects Onsager symmetry. The singularity of the Onsager matrix corresponds to the so-called tight coupling limit^62,63^, for which the strict proportionality between the heat and magnon currents occurs. This feature leads to far-reaching consequences that will be described in the next section. Spin conductance derived for arbitrary *U* is presented in the Supplementary Information.

## Results and discussion

### Spin Seebeck and spin Peltier effects

Defining spin Seebeck coefficient as spin voltage drop generated by temperature difference under condition of vanishing spin current one obtains;

17 $$\begin{aligned} S_s\equiv -\left( \frac{\Delta \mu _s}{\Delta T}\right) _{J_s=0}=\frac{g\mu _BB}{T}. \end{aligned}$$

In turn, spin Peltier coefficient is defined as ratio of heat current to spin current under condition of vanishing temperature bias;

18 $$\begin{aligned} \pi _s\equiv -\left( \frac{J_Q}{J_s}\right) _{\Delta T=0}=S_sT=g\mu _BB. \end{aligned}$$

Note that both spin Seebeck and Peltier coefficients acquire the above forms disregarding the value of parameter *U* i.e. $S_s$ and $\pi _s$ are described by the same formulas for finite *U* and for $U\rightarrow \infty$ cases.

Both $S_s$ and $\pi _s$ are functions of energy transferred by magnon ($\varepsilon _{\downarrow }-\varepsilon _{\uparrow }=g\mu _BB$) and don’t depend on dot’s level position. Especially, in the case of the latter coefficient the dependence on magnon energy is physically clear as it is equal to energy exchanged between external leads. It clearly shows how much heat is carried per unit particle (magnon). Moreover, spin Seebeck and spin Peltier coefficients are directly related with each other resembling the same symmetry between corresponding coefficients of the conventional thermoelectric phenomena. In the case of spin counterparts of thermoelectric effects, the spin Peltier phenomenon can be regarded as the back-action of the spin Seebeck effect i.e. the spin Seebeck effect will drive a spin current which by means of spin Peltier effect will transfer the heat from the hot to the cold junction.

In turn, the spin Seebeck coefficient is proportional to energy carried by magnon and inversely proportional to the temperature. Zero temperature limit should be regarded carefully as no magnons can be created and thus the spin Seebeck coefficient vanishes as temperature tends to zero. However, this case is excluded as we assumed that $\Delta T\ll T$. Note also that utilized here master equation method requires condition $k_BT\gg \Gamma$, and thus, the results are reliable only when the condition is fulfilled. The temperature dependence of spin Seebeck coefficient leads to high values of $S_s$ for low temperature regime i.e. for $k_BT\ll g\mu _BB$, which means that one has to apply a relatively large spin bias voltage to compensate thermally-induced spin current. In turn, for higher temperatures it is easier to compensate thermally-induced spin current as the spin Seebeck coefficient decreases with increasing temperature. This feature is a consequence of the competition between Bose–Einstein and Fermi–Dirac distributions. On the one hand, the number of magnons in the magnetic insulator reservoir grows with increasing temperature and one naively expects that more magnons can be transferred through the system. On the other hand, smearing the Fermi distribution around the Fermi level as temperature grows leads to a decreasing rate of tunneling electrons through the junction between QD and metallic lead.

**Figure 2:**
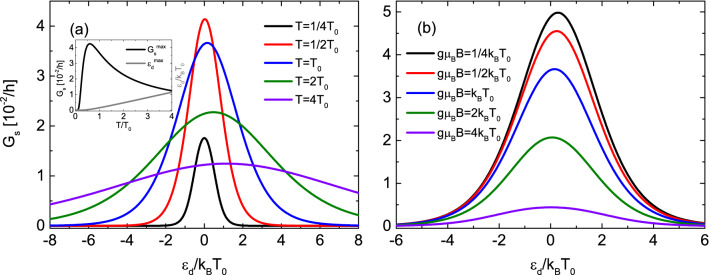
Spin conductance in the limit of $U\rightarrow \infty$. Spin conductance as a function of the dot’s level position calculated for indicated values of (**a**) temperature and for $g\mu _BB=k_BT_0$ and (**b**) the applied magnetic field and for $k_BT=k_BT_0$. Inset shows temperature dependence of dot’s energy level (grey) for which spin conductance is maximal and corresponding maximal value of $G_s$ (black). The other parameters are: $\Gamma _e=\Gamma _m=0.1k_BT_0$ and $k_BT_0=0.1$ meV.

### Heat conductance

Defining heat conductance as ratio of heat current to temperature bias under condition of vanishing spin current;

19 $$\begin{aligned} \kappa =\left( \frac{J_Q}{\Delta T}\right) _{J_s=0} \end{aligned}$$

one quickly concludes that $\kappa =0$ for both finite *U* and $U\rightarrow \infty$ cases as a consequence of the tight coupling limit. This will lead to the figure of merit $Z_sT\equiv G_sS_s^2/\kappa \rightarrow \infty$ indicating that the device works at Carnot efficiency (see Supplementary Information for the proof). This is a straightforward consequence of vanishing heat current as spin current is assumed to be zero [compare Eq. (12) with Eq. (13)]. In other words, when the build-up spin bias $\Delta \mu _s$ is induced by temperature difference $\Delta T$ it compensates both spin and heat currents. This phenomenon is in strong opposition to the case of purely electric system with both electrodes being reservoirs of electrons, where vanishing of charge current doesn’t imply vanishing of heat current, i.e. a flux of electrons flowing from hot reservoir to cold one transfers higher energy than the same flux of electrons flowing from cold to hot electrode which leads to finite heat conductance. One should note that in real systems phonons transfer the energy and will contribute to thermal conductance. Hence, the lattice thermal conductance will remove the infinity of *ZT* although it may still be large. However, one should remember that *ZT* is linear response quantity which characterizes the device’s performance close to zero power and gives only a little insight outside the linear response regime. Usually, $ZT\rightarrow \infty$ does not give maximal efficiency at finite power output. Moreover, when the Carnot efficiency is achieved the system must be reversible and then usually the power output vanishes^64^.

Finally, introducing above defined transport coefficients, Eq. (15) can be rewritten as;

20 $$\begin{aligned} \left( \begin{array}{c} J_s \\ J_Q \\ \end{array} \right) = \left( \begin{array}{cc} G_s &{} G_s S_s \\ G_s\pi _s &{} G_s S_s\pi _s \\ \end{array} \right) \left( \begin{array}{c} \Delta \mu _s \\ \Delta T \\ \end{array} \right) , \end{aligned}$$

which also clearly shows that heat conductance $\kappa$ vanishes.

**Figure 3:**
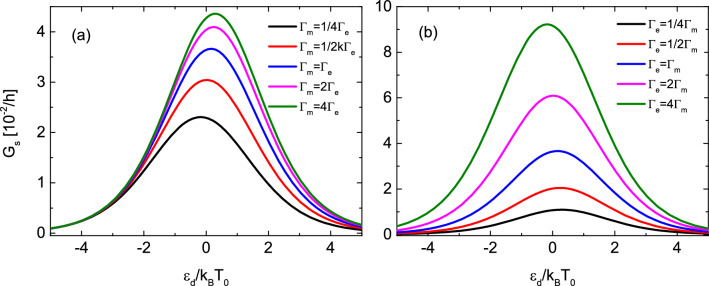
Spin conductance for asymmetric couplings in the limit of $U\rightarrow \infty$. Spin conductance as a function of the dot’s level position calculated for indicated values of (**a**) coupling strengths to magnonic reservoir with $\Gamma _e=0.1k_BT_0$ (**b**) coupling strengths to electronic reservoir with $\Gamma _m=0.1k_BT_0$. The other parameters are: $g\mu _BB=k_BT_0$, $k_BT=k_BT_0$ and $k_BT_0=0.1$ meV.

### Spin conductance

#### Limit of $U\rightarrow \infty$

As the spin conductance (16) acquires a more complex form we calculated it numerically for various sets of parameters and presented the obtained results graphically. In Fig. 2 we show spin conductance dependence on the dot’s level position for indicated values of temperature (a) and applied magnetic field (b) and calculated under condition of infinitely large ondot Coulomb repulsion ($U\rightarrow \infty$). For the sake of simplicity we assumed that $\mu =0$ and $\Gamma _e=\Gamma _m$. First of all, one can notice that the spin conductance is not symmetric with respect to zero dot’s level position which can be attributed to the fact that the two leads are of different type, one is fermionic and the other one is bosonic. Generally, the position of the maximum in conductance is a function of both applied magnetic field and the temperature and can be found from the formula;

21 $$\begin{aligned} \varepsilon _d^{max}=k_BT\ln \left[ \frac{\sqrt{2\Gamma _m}e^{x}\sqrt{1+e^{x}}}{\sqrt{\Gamma _m-\Gamma _e+(\Gamma _m+\Gamma _e)e^{x}}}\right] \end{aligned}$$

with $x=g\mu _BB/k_BT$. One can deduce that for symmetric coupling, $\Gamma _e=\Gamma _m$, and for finite temperature the maximum is situated at the positive value of dot’s level position. Moreover, with increasing temperature (for given magnetic field) the maximum of conductance moves away from zero to positive values of dot’s level energy and simultaneously the width of the peak of the spin conductance grows. The last feature results from the temperature dependence of the Fermi function. The intensity of spin conductance is a function of both the temperature and the applied magnetic field i.e. energy of the magnon. Figure 2a and the inset show that maximum of the peak is nonmonotonic function of temperature. Firstly, it grows with increasing the temperature and after reaching maximal value at certain temperature it decreases with further increase of temperature. In turn, when increasing the magnetic field the maximum of spin conductance monotonically decreases as shown in Fig. 2b. This behavior follows directly from the Bose-Einstein distribution function, which leads to a decrease of magnons’ density with increasing magnetic field and consequently to lower transmission of the magnons.

In turn, temperature dependence of the spin conductance results rather from the competition between Bose–Einstein and Fermi–Dirac distributions, similarly as temperature dependence of spin Seebeck effect explained earlier. An increase of temperature leads to enhancement of density of magnons in the magnetic insulator electrode and simultaneously it smears the Fermi distribution around the Fermi level. As a result, for low temperatures there are not many magnons and consequently small magnon current is flowing, and hence, small spin conductance. For higher temperatures more magnons are excited in the magnonic reservoir, and thus, larger spin conductance is noticed. However, further increase of temperature leads to decrease of transmitted magnons despite its increasing density in the magnonic reservoir. This effect can be understood by looking at the temperature dependence of the Fermi distribution. For sufficiently high temperature the distributions of electrons (in the metallic electrode) with energies $\varepsilon =\varepsilon _\downarrow$ and $\varepsilon =\varepsilon _\uparrow$ differ only a little. Thus, the probability of tunneling of an electron with spin $\sigma$ to or from the metallic electrode becomes more and more similar with increasing temperature which leads to suppressions of charge currents in both spin channels and consequently spin current becomes diminished.

Furthermore, the width of the peak rather weakly depends on the magnetic field—it slowly grows with increasing *B*. Moreover, the position of the maximum of the spin conductance moves to lower values of dot’s energy level with increasing the magnetic field (at constant temperature) oppositely to the temperature dependence described above.

**Figure 4:**
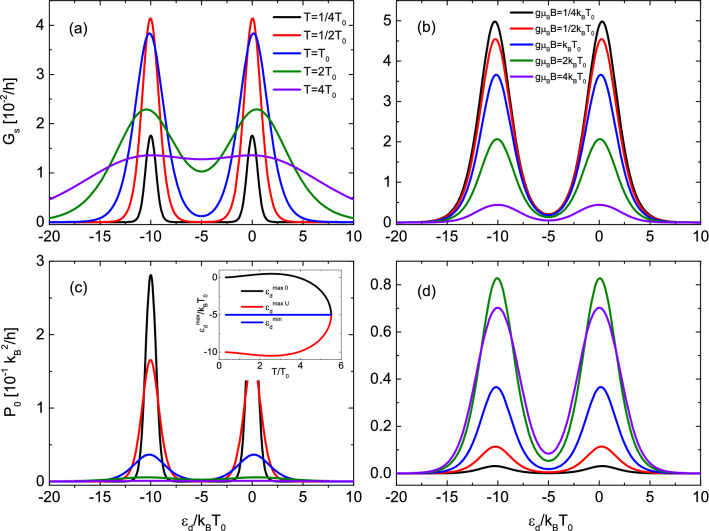
Spin conductance and spin power factor in the case of finite *U*. Spin conductance and corresponding power factor as a function of the dot’s level position calculated for indicated values of temperature (**a**) and (**c**) and for $g\mu _BB=k_BT_0$ and the applied magnetic field (**b**) and (**d**) and for $k_BT=k_BT_0$ . Inset shows temperature dependence of dot’s energy level for which spin conductance achieves maximum (black and red) and minimum (blue). The other parameters are: $U=10k_BT_0$, $\Gamma _e=\Gamma _m=0.1k_BT_0$ and $k_BT_0=0.1$ meV.

In Fig. 3 we present spin conductance dependence on the dot’s level position calculated for different values of (a) [(b)] coupling strengths to magnonic [electronic] reservoir with constant coupling to electronic [magnonic] one. One can notice that the width of the resonance in spin conductance only weakly depends on coupling to the magnonic reservoir and up to value $\Gamma _m=2\Gamma _e$ is almost constant. For larger values of $\Gamma _m$, i.e. for $\Gamma _m>2\Gamma _e$, small increase of the width can be observed. In turn, the width of the peak becomes larger with increasing $\Gamma _e$. On the other hand, intensity of spin conductance grows monotonically with increasing any of the couplings due to enhancement of magnon and electron tunneling rates.

Moreover, one can notice that for asymmetric couplings the conductance’s maximum can be situated at positive or negative dot’s level energies depending on the ratio $\Gamma _m/\Gamma _e$. Specifically, when $\Gamma _m/\Gamma _e>\tanh {(g\mu _B B/2k_BT)}$ the maximum occurs for positive values of dot’s energy level, whereas for $\Gamma _m/\Gamma _e<\tanh {(g\mu _B B/2k_BT)}$ it is located at negative values of $\varepsilon _d$. When the equality holds, $\Gamma _m/\Gamma _e=\tanh {(g\mu _B B/2k_BT)}$, spin conductance becomes symmetric with respect to $\varepsilon _d=0$. Thus, for a given ratio of couplings $\Gamma _m/\Gamma _e$ one can obtain this symmetry by properly tuning the ratio *B*/*T*. Inversely, when the *B*/*T* ratio is set, the symmetry can be recovered by proper selection of $\Gamma _m/\Gamma _e$ ratio.

#### Case of finite *U*

In this section we consider an influence of finite intradot Coulomb repulsion on spin thermoelectric coefficients. In Fig. 4 spin conductance dependence on the dot’s level position for indicated values of temperature (a) and applied magnetic field (c) is presented. The main difference in respect to the $U\rightarrow \infty$ case, presented in Fig. 2, is a double peak structure. One peak in spin conductance is associated with resonance at $\varepsilon _d\approx 0$, whereas the second maximum appears in the vicinity of $\varepsilon _d=-U$. The latter peak is present only for finite *U* values. The minimum between the maxima is located at $\varepsilon _d^{min}=-U/2+\mu /k_BT$, and thus, assuming $\mu =0$ one obtains $\varepsilon _d^{min}=-U/2$. Moreover, the intensities of maxima in the spin conductance follow the same behavior with changing temperature and applied magnetic field as those calculated for the $U\rightarrow \infty$ case. However, here the positions of maxima exhibits slightly different behavior than for the $U\rightarrow \infty$ case. Specifically, at low temperature limit the positions of the maxima are located at $\varepsilon _d^{0 max}\gtrsim 0$ and $\varepsilon _d^{U max}\lesssim -U$. Furthermore, with increasing temperature the maxima move away from each other until the temperature reaches the critical value $T_{c1}$ which for assumed parameters and for $g\mu _BB=k_BT_0$ equals to $T_{c1}/T_0\approx 2.564$. For this temperature the separation between the maxima is the largest. Further increase of temperature leads to shrinking of the separation and for certain temperature $T_{c2}$ the maxima occur for $\varepsilon _d^{0 max}=0$ and $\varepsilon _d^{U max}=-U$ i.e. when $T_{c2}/T_0\approx 3.881$ for $g\mu _BB=k_BT_0$. For temperature $T>T_{c2}$ the positions of both maxima become negative and move closer to each other. Finally, both maxima merge into one maximum which occurs for temperature $T_{c3}$ ($T_{c3}/T_0\approx 5.506$ for $g\mu _BB=k_BT_0$).

In Fig. 4c and d we show spin power factor corresponding to spin conductance displayed in Fig. 4a and b, respectively. The power factor is defined as;

22 $$\begin{aligned} P_0=G_sS_s^2 \end{aligned}$$

and determines the effectiveness of heat to spin current conversion in the linear response regime. The power factor is symmetric with respect to the particle-hole point given by $\varepsilon _d=-U/2$. One can notice that the power factor achieves large values in the low temperature regime and drops with increasing temperature owing to temperature dependence of spin Seebeck coefficient [see Eq. (17)]. In turn, the power factor is a nonmonotonic function of the applied magnetic field. For sufficiently low or sufficiently large magnetic fields it becomes suppressed, whereas for moderate magnetic fields the power factor achieves maximal values, which follows from peculiar dependence of spin conductance and spin thermopower on magnetic field.

## Conclusions

In summary, we have analyzed spin thermoelectric properties of a quantum dot coupled to a metallic electrode and magnetic insulator. We have considered two cases: with infinite intradot Coulomb repulsion ($U\rightarrow \infty$) and with finite values of *U*. In both cases the spin Seebeck and spin Peltier coefficients acquire the same forms and don’t depend on dot’s level position. We provided analytical formulas for these coefficients which showed that spin Seebeck coefficient depends on temperature and applied magnetic field, whereas spin Peltier coefficient equals the energy carried by a magnon. We have also shown that spin Seebeck and spin Peltier coefficients are related via Onsager reciprocal relation. Additionally, we have shown that in the considered system heat conductance vanishes which means that the system works at Carnot’s efficiency.

Furthermore, we have analyzed in detail spin conductance dependence on different system’s parameters regarding both infinite-*U* and finite-*U* cases separately. Moreover, by introducing spin power factor we have been able to indicate conditions under which the system works more effectively.

## Supplementary Information

Supplementary Information.
