# Supplementary material for: Spin-thermoelectric effects in a quantum dot hybrid system with magnetic insulator
Source: Sci Rep. 2022 Mar 30;12:5348. doi: 10.1038/s41598-022-09105-z (PMC8969188; doi:10.1038/s41598-022-09105-z)
Supplement: Supplementary file 1 — Supplementary Information. [file 41598_2022_9105_MOESM1_ESM.pdf]

Supplementary Information for: Spin-thermoelectric effects in a quantum dot hybrid system with magnetic insulator

Piotr Trocha[1,*] **and Emil Siuda**[1]

[1]Institute of Spintronics and Quantum Information, Faculty of Physics, Adam Mickiewicz University, Poznań, 61-614, Poland
[*]ptrocha@amu.edu.pl

Spin conductance for finite U

Spin conductance formula for finite U value acquires the following form;

$$G_s = \frac{\Gamma_e \Gamma_m \mathscr{F}_{1\mu}\left[\mathscr{F}_0 + \mathscr{F}_\uparrow + \mathscr{F}_\downarrow + \mathscr{F}_1\right]}{k_B T \mathscr{A}\left[\Gamma_m \mathscr{B} + (\Gamma_m - \Gamma_e)\mathscr{C} + (\Gamma_m + \Gamma_e)\mathscr{D} + (\frac{1}{2}\Gamma_m - \Gamma_e)\mathscr{F}_{2\uparrow} + (\frac{1}{2}\Gamma_m + \Gamma_e)\mathscr{F}_{2\downarrow}\right]} \tag{1}$$

with $\mathscr{A} = \mathscr{F}_0 + \mathscr{F}_{\uparrow U} + \mathscr{F}_{\downarrow U} + \mathscr{F}_1$, $\mathscr{B} = \mathscr{F}_{\uparrow\downarrow} + 2\mathscr{F}_{1\mu}$, $\mathscr{C} = \mathscr{F}_{\uparrow 2\mu} + \mathscr{F}_2$, $\mathscr{D} = \mathscr{F}_{\downarrow 2\mu} + \mathscr{F}_3$, and $\mathscr{F}_0 = \mathscr{F}(\mu)$, $\mathscr{F}_\uparrow = \mathscr{F}(\varepsilon_\uparrow + \mu)$, $\mathscr{F}_\downarrow = \mathscr{F}(\varepsilon_\downarrow + \mu)$, $\mathscr{F}_{\uparrow 2\mu} = \mathscr{F}(\varepsilon_\uparrow + 2\mu)$, $\mathscr{F}_{\downarrow 2\mu} = \mathscr{F}(\varepsilon_\downarrow + 2\mu)$, $\mathscr{F}_{\uparrow\downarrow} = \mathscr{F}(\varepsilon_\uparrow + \varepsilon_\downarrow + \mu)$, $\mathscr{F}_{2\uparrow} = \mathscr{F}(2\varepsilon_\uparrow + \mu)$, $\mathscr{F}_{2\downarrow} = \mathscr{F}(2\varepsilon_\downarrow + \mu)$, $\mathscr{F}_{\uparrow U} = \mathscr{F}(\varepsilon_\uparrow + U + \mu)$, $\mathscr{F}_{\downarrow U} = \mathscr{F}(\varepsilon_\downarrow + U + \mu)$, $\mathscr{F}_1 = \mathscr{F}(\varepsilon_\uparrow + \varepsilon_\downarrow + U)$, $\mathscr{F}_{1\mu} = \mathscr{F}(\varepsilon_\uparrow + \varepsilon_\downarrow + U + \mu)$, $\mathscr{F}_2 = \mathscr{F}(2\varepsilon_\uparrow + \varepsilon_\downarrow + U)$, $\mathscr{F}_3 = \mathscr{F}(2\varepsilon_\downarrow + \varepsilon_\uparrow + U)$.

Remarks on efficiency

Let us rewrite linear response equations for magnon (spin) and heat currents, i. e. Eq. (20) displayed in the manuscript, by introducing relevant Onsager coefficients $\mathscr{L}_{ij}$ $(i,j = 1,2)$ and generalized forces $\mathscr{X}_s = \Delta\mu_s/T$, $\mathscr{X}_T = \Delta T/T^2$;

$$\begin{pmatrix} J_m \\ J_Q \end{pmatrix} = \begin{pmatrix} \mathscr{L}_{11} & \mathscr{L}_{12} \\ \mathscr{L}_{21} & \mathscr{L}_{22} \end{pmatrix} \begin{pmatrix} \Delta\mu_s/T \\ \Delta T/T^2 \end{pmatrix}, \tag{2}$$

with $\mathscr{L}_{11} = TG_s$, $\mathscr{L}_{12} = \mathscr{L}_{21} = G_s S_s T^2$, $\mathscr{L}_{22} = (\kappa + G_s S_s^2 T)T^2$. It was shown above that $\kappa = 0$ but we keep it arbitrary and we will take the limit $\kappa \to 0$ at the end of the considerations. Inverting the above relations one can express spin thermoelectric coefficients by means of Onsager coefficients;

$$G_s = \frac{\mathscr{L}_{11}}{T} \tag{3}$$

$$S_s = \frac{1}{T}\frac{\mathscr{L}_{12}}{\mathscr{L}_{11}} \tag{4}$$

$$\kappa = \frac{1}{T^2}\frac{\mathscr{L}_{11}\mathscr{L}_{22} - \mathscr{L}_{12}\mathscr{L}_{21}}{\mathscr{L}_{11}} \tag{5}$$

and $\pi_s = \mathscr{L}_{21}/\mathscr{L}_{11}$ for completeness.

The efficiency of heat to work conversion is defined as;

$$\eta_s = \frac{P_s}{J_Q} \tag{6}$$

with $P_s = -J_m \Delta\mu_s$ being output spin power. In the linear response the efficiency reads;

$$\eta_s = \frac{-T\mathscr{X}_s(\mathscr{L}_{11}\mathscr{X}_s + \mathscr{L}_{12}\mathscr{X}_T)}{\mathscr{L}_{21}\mathscr{X}_s + \mathscr{L}_{22}\mathscr{X}_T} \tag{7}$$

Maximizing η_s with respect to generalized force $\mathscr{X}_s$ for fixed $\mathscr{X}_T$ i. e. with respect to $\Delta\mu_s$ with fixed ΔT, gives maximum efficiency;

$$\eta_s^{max} = \eta_C \frac{\sqrt{Z_s T + 1} - 1}{\sqrt{Z_s T + 1} + 1} \tag{8}$$

with $Z_sT = G_sS_s^2T/\kappa$ being the figure of merit and $\eta_C = 1 - T_e/T_m$ denoting Carnot efficiency which in the linear response theory takes the form $\eta_C = \Delta T/T$. For the considered system $\kappa = 0$, and thus, $ZT \to \infty$ which leads to $\eta_s^{max} \to \eta_C$.

Maximizing output spin power P_s with respect to $\mathscr{X}_s$ for fixed $\mathscr{X}_T$ one obtains maximum spin power[1];

$$P_s^{max} = \frac{1}{4}G_sS_s^2(\Delta T)^2. \tag{9}$$

Inserting Eq.(9) into Eq.(6) together with $\mathscr{X}_s$ which maximizes P_s leads to formula for efficiency at maximum power;

$$\eta_s(P_s^{max}) = \frac{\eta_C}{2}\frac{Z_sT}{Z_sT+2} \tag{10}$$

which in the limit $ZT \to \infty$ tends to $\eta_s(P_s^{max}) \to \frac{\eta_C}{2}$. Thus, efficiency at maximum power is half of maximum efficiency, which coincides with the linear response expansion of the Curzon-Ahlborn efficiency[2].

References

1. Benenti, G., Casati, G., Saito, K. & Whitney, R. S. Fundamental aspects of steady-state conversion of heat to work at the nanoscale. *Phys. Rep.* **694**, 1–124, 10.1016/j.physrep.2017.05.008 (2017).

2. Curzon, F. L. & Ahlborn, B. Efficiency of a Carnot engine at maximum power output. *Am. J. Phys.* **43**, 22–24, 10.1119/1.10023 (1975).
